# The impact of short-term non-steroidal androgen antagonist therapy on PSMA expression and tumor cellularity studied with dynamic [^68^Ga]Ga-PSMA-11 PET/MR in hormone-sensitive prostate cancer patients, a preliminary longitudinal prospective study

**DOI:** 10.1186/s13550-025-01328-1

**Published:** 2025-10-02

**Authors:** Alejandro Sanchez-Crespo, Olof Jonmarker, Fredrik Jäderling, Stefan Carlsson, Mats Olsson, Chunde Li, Rimma Axelsson

**Affiliations:** 1 Department of Nuclear Medicine and Hospital Physics, Medical Diagnosis Karolinska, Karolinska University Hospital, Stockholm, Sweden; 2 Department of Molecular Medicine and Surgery, Karolinska Institutet, Stockholm, Sweden; 3 Department of Clinical Science, Intervention and Technology, Karolinska Institutet, Stockholm, Sweden; 4 Department of Radiology, Capio S:t Görans Hospital, Stockholm, Sweden; 5 Department of Urology, Tema Cancer, Karolinska University Hospital, Stockholm, Sweden; 6 Department of Clinical Science and Education, Karolinska Institutet, Stockholm, Sweden; 7 Department of Oncology, Södersjukhuset, Stockholm, Sweden; 8 Theranostics Trial Center, Karolinska University Hospital, Stockholm, Sweden

## Abstract

**Background:**

Prostate-specific membrane antigen (PSMA) is overexpressed in most prostate cancers (PCa) and is targeted in both diagnostic and therapeutic applications. Preclinical studies suggest that short-term androgen blockade may upregulate PSMA expression, potentially enhancing lesion detectability with [^68^Ga]Ga-PSMA-11 positron emission computed tomography (PET) and the therapeutic efficacy of PSMA-targeted radioligands. However, clinical data remains limited and inconsistent. The aim of this study was to assess the impact of short-term non-steroidal androgen blockade therapy (NSAA) on lesion PSMA expression and cellularity using dynamic [^68^Ga]Ga-PSMA-11PET and diffusion-weighted magnetic resonance imaging (MR) for simultaneous estimation of binding potential (BP_ND_) and apparent diffusion coefficient (ADC) in hormone-naïve patients with high-risk PCa without bone metastases.

**Results:**

A significant serum prostate specific antigen (PSA) decline was observed in 7/8 patients (median PSA fold change − 88.3% at day 28), indicating positive biochemical response since the NSAA start. Among the observed eight lesions with detectable [⁶⁸Ga]Ga-PSMA-11 uptake, seven exhibited a non-linear and non-monotonic longitudinal trajectory of BP_ND_, characterized by a rebound during the mid-treatment phase. In contrast, ADC values progressively increased from baseline for all lesions, suggesting reduced tumour cellularity as treatment progresses. Static SUV measurements poorly reflected these dynamic changes in PSMA expression, indicating limited sensitivity.

**Conclusion:**

Short-term NSAA induces transient PSMA upregulation in hormone-sensitive PCa lesions despite declining cellularity, which may support its cointegration to PSMA-targeted therapies for this population.

**Supplementary Information:**

The online version contains supplementary material available at 10.1186/s13550-025-01328-1.

## Introduction

Prostate-specific membrane antigen (PSMA) is a transmembrane protein commonly expressed in prostate cancers (PCa), with levels generally correlating with tumour aggressiveness [1]. Notably, PSMA expression can be reduced in very advanced or poorly differentiated PCa [2]. This differential expression has established PSMA as a critical target for both diagnostic and therapeutic radioligands in contemporary clinical oncology. Among these, PSMA-targeted positron emission tomography (PET) using radioligands such as [⁶⁸Ga]Ga-PSMA-11 has become a routine modality for staging both primary and recurrent PCa globally. Preclinical studies have demonstrated the high sensitivity of [⁶⁸Ga]Ga-PSMA PET, which can detect submillimeter clusters of PCa cells, exceeding the conventional spatial resolution limits of the PET scanner [3]. Clinically, the integration of PSMA-PET with magnetic resonance imaging (MR) has yielded detection sensitivities of up to 95% [4]. In this combined modality, diffusion-weighted imaging (DWI) and the derived apparent diffusion coefficient (ADC) maps are essential, with ADC serving as a surrogate biomarker for tumour cellularity [5, 6]. In vitro and in vivo studies have consistently shown that short-term androgen deprivation therapy (ADT) can upregulate PSMA expression [7–10]. This finding has led to the hypothesis that short-term androgen blockade may enhance both the diagnostic sensitivity of PSMA-PET and the therapeutic efficacy of PSMA-targeted radioligands such as [¹⁷⁷Lu]Lu-PSMA [11]. These effects may be particularly advantageous in patients with primary PCa or biochemical recurrence, where improved lesion detection could directly impact clinical decision-making.

A recent phase 2 trial demonstrated that combining [¹⁷⁷Lu]Lu-PSMA-617 with docetaxel resulted in superior early treatment responses compared to docetaxel monotherapy in patients with metastatic hormone-sensitive prostate cancer [12]. However, androgen blockade was not administered concurrently with [¹⁷⁷Lu]Lu-PSMA in that study. Furthermore, earlier prospective clinical studies evaluating PSMA modulation by non-steroidal antiandrogens (NSAAs) have produced inconsistent results [13–20]. While increased PSMA expression was more frequently observed in bone metastases of castration-resistant PCa, lesion-specific heterogeneity was a consistent finding. Importantly, no study reported a loss of lesion detectability following short-term androgen blockade, suggesting that such short-term treatment does not compromise diagnostic performance. These inconclusive findings may be attributed to several methodological limitations, including:

1. Reliance on standardized uptake values (SUV) from static PET imaging as indirect measures of PSMA expression. SUV are influenced by multiple confounding factors—such as lesion cellularity, receptor density, unspecific background binding, tracer pharmacokinetics, and clearance—and do not directly quantify receptor availability.
2. Insufficient consideration of baseline PSMA expression and concurrent changes in lesion cellularity.
3. Inclusion of heterogeneous patient populations treated with varying types and durations of hormonal therapy.

In this prospective interventional longitudinal clinical study, we aimed to address these limitations by utilizing dynamic [⁶⁸Ga]Ga-PSMA-11 PET in conjunction with diffusion-weighted MR. This approach enabled simultaneous assessment of changes in PSMA expression—quantified via non-displaceable binding potential (BP_ND_)—and lesion cellularity—assessed via ADC. The BP_ND_ is a dimensionless parameter derived from kinetic modelling that reflects the density of available PSMA binding sites relative to non-specific binding. Our primary objective was to investigate the effects of short-term androgen blockade on baseline lesion levels of BP_ND_ and ADC. Specifically, we addressed the following research questions:

1. Does short-term NSAA therapy influence radiologic staging with [⁶⁸Ga]Ga-PSMA-11 PET/MR in high-risk, hormone-sensitive PCa patients without bone metastases?
2. Does short-term NSAA therapy induce measurable changes in lesion-levels of PSMA receptor expression and cellularity over time?
3. Are changes in ADC and duration of NSAA therapy independent predictors of PSMA modulation?

## Materials and methods

### Study design and patient selection

**Fig. 1:**
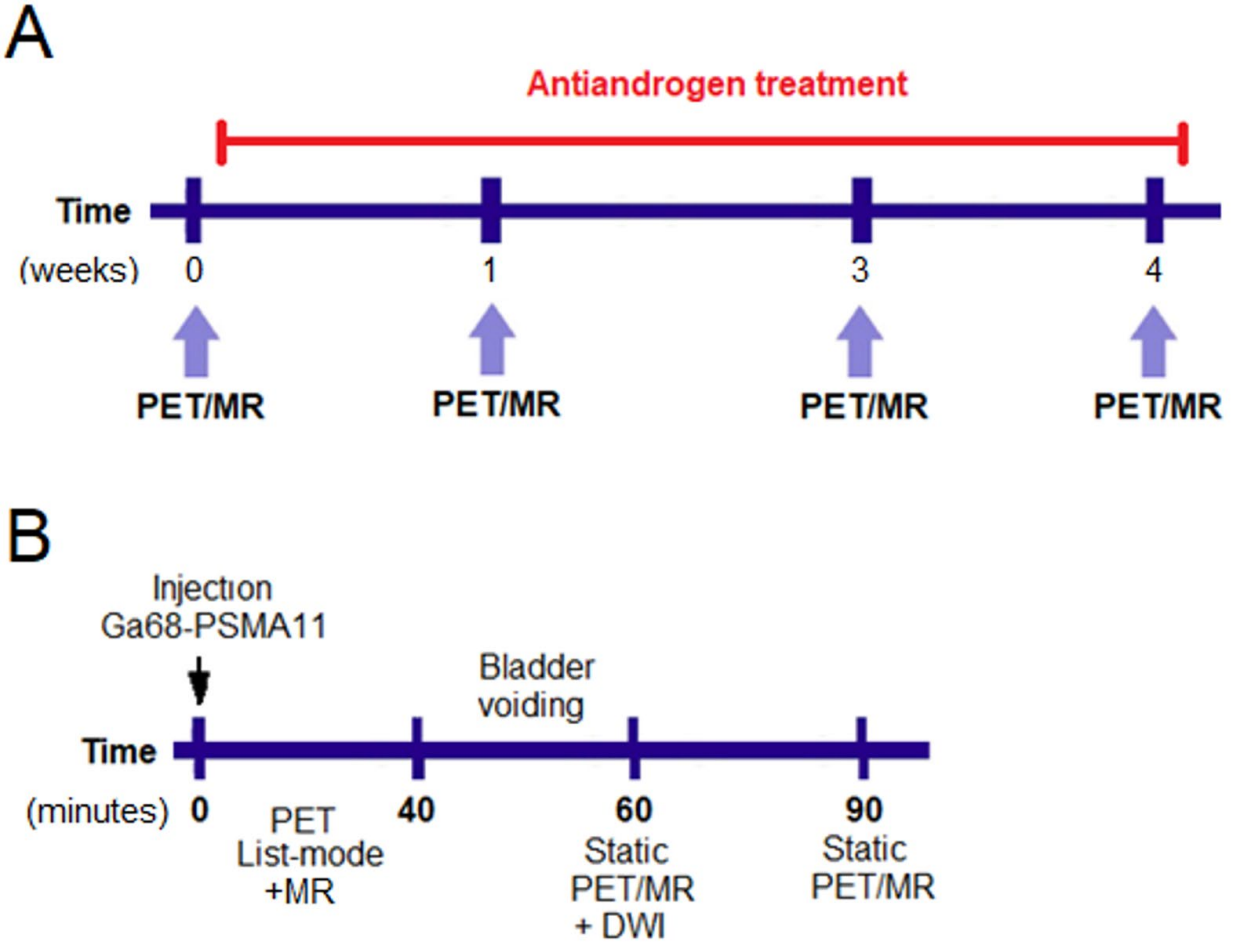
(**A**) Study design illustrating the frequency of hospital visits from baseline throughout the course of antiandrogen treatment. (**B**) Schematic overview of the [^68^Ga]Ga-PSMA-11PET/MR imaging acquisition protocol conducted at each visit. Abbreviations: DWI = Diffusion weighted imaging

This is an interventional prospective longitudinal study conducted in conformance with the latest revision of Declaration of Helsinki of medical research and approved by the Swedish Ethical Review Authority (Registration number 2019–03605). All patients signed a written informed consent prior to enrolment. Patients were enrolled in the study between September 2019 and March 2022, thereafter the study was closed because of a low enrolment rate. The inclusion criteria were patients with biochemical relapse or newly diagnosed primary prostate cancer, without bone metastasis as verified by[^99m^Tc]Tc-HDP bone scintigraphy and with no prior hormonal treatment (hormone-naïve). All patients were treated according to current clinical routine with Bicalutamide (150 mg daily), a first-generation nonsteroidal antiandrogen agent (NSAA). The study subjects were serially examined using a 3 tesla GE Signa PET-MR system with a 25 cm axial field of view (General Electric Healthcare), at baseline and after one, three, and four weeks of Bicalutamide treatment, Fig. 1-A. Serum prostate specific antigen (PSA) level was monitored in conjunction to every PET imaging session. At every examination day the PET/MR scanning protocol was performed as illustrated in Fig. 1-B. Briefly, 2 MBq [^68^Ga]Ga-PSMA-11per kg body weight was intravenously administered as a bolus injection and a PET list mode acquisition was simultaneously started in conjunction with T2 and LAVA-Flex MR sequences for lesion localization and PET image attenuation correction. The PET list-mode acquisition was performed over the pelvic region and acquired for 40 min. After that, the participants were allowed to exit the investigation room to empty their urinary bladder. Thereafter static PET (4 min per bed position) - MR acquisitions (including T2 and LAVA-Flex) from skull base to mid-thigh were acquired at 60- and 90-minutes post tracer administration. The static PET/MR scan at 60 min also included a diffusion weighted MR sequence (DWI) of the pelvis. Directly after the study intervention at week 4, all the patients in this study began standard-of-care treatment with either radiotherapy or surgery.

### Visual assessment of the static PET/MR scans for disease re-staging

For all patients and visits to the hospital, the static PET/MR images obtained at 60 -minutes post tracer administration, were visually reviewed for disease restaging according to clinical routine standards jointly and unblinded by the experienced radiologists and nuclear medicine physicians of our research group.

### [^68>^Ga]Ga-PSMA-11 PET/MR image quantification

All PET images were iteratively reconstructed using an ordered subset expectation maximization algorithm with 28 subsets, 2 iterations, 5-mm gaussian filter, time-of-flight correction and photon scattering and attenuation correction using the LAVA-MR pulse sequence. The PET list-mode acquisition was reconstructed in 55 timeframes with the following time sequence: 15 frames x 20 s, 15 frames x 40 s and 25 frames x 60 s. At each imaging session (Fig. 1B), all acquired PET/MR images were decay corrected to the time for tracer administration. For each patient and hospital visit, the dynamic and the 60 min PET/MR datasets were rigidly aligned to the 90-minute acquisition using the automatic registration tool in Affinity 3.0.4 (Hermes Medical Solutions, Stockholm, Sweden). This ensured consistent positioning of the volume of interest (VOI) across all scans for time–activity curve (TAC) extraction. To account for changes in the lesion volume on PSMA-PET during the study period and to minimize partial volume effects, an adaptive segmentation approach with a 40% relative threshold [21], was used to delineate each lesion in the static PET image acquired at 90 min at every imaging session. The lesions TAC in Becquerel per millilitre (Bq/ml) from 0 to 90 min post tracer administration were then generated at every patient visit from this VOI. The BP_ND_ for each lesion was then estimated using the simplified reference tissue model [22, 23]. For this model, the reference tissue TAC was generated from a manually drawn VOI in the 90 min T2-MR image covering the gluteus maximus (which is devoid of PSMA receptors). Non-linear fitting was performed in PMOD software v4.2 (PMOD Technologies Ltd, Zürich, Switzerland) using the Marquardt fitting method and relative weighting to PET frame duration and counts. The mean ADC for each lesion was calculated from the diffusion-weighted MR images using a mono-exponential model. Additionally, and in accordance with our clinical routine, peak SUV values (SUVpeak) were obtained from the 60 min post tracer administration PET images at every patient visit to the hospital. SUVpeak was defined as the maximum SUV average within a 1 ml sub-volume of the lesion VOI. The relative longitudinal fold changes from baseline values of BP_ND_, ADC, plasma-PSA and SUVpeak (denoted as ΔBP_ND_%, ΔADC%, ΔPSA% and ΔSUV%, respectively) were calculated by subtracting each follow-up value from baseline value and dividing by the baseline value.

#### Data analysis

Descriptive statistics for the measured variables are reported as medians with interquartile ranges (IQRs). Linear mixed-effects (LME) models, incorporating random effects to account for patient-specific variability, were used to investigate the temporal evolution of each outcome variable over time in response to NSAA treatment. A separate extended LME model was used to examine whether changes in lesion PSMA expression (ΔBP_ND_%) were associated with changes in tissue cellularity (ΔADC%) over time during NSAA treatment. Additionally, an LME model was applied to assess the agreement between two methods of quantifying PSMA receptor expression—ΔSUV% from static imaging and ΔBP_ND_% from dynamic imaging—by estimating the bias and limits of agreement. The generalized concordance correlation coefficient between these methods was also computed [24]. Statistical significance of fixed effects in the LME models was evaluated using analysis of variance (ANOVA). All statistical analyses were performed using MATLAB version 9.13.0 (R2022b) (The MathWorks Inc., Natick, MA, USA). LME models were fitted using restricted maximum likelihood estimation with the quasi-Newton optimization algorithm. A two-tailed p-value < 0.05 was considered statistically significant.

## Results

**Fig. 2:**
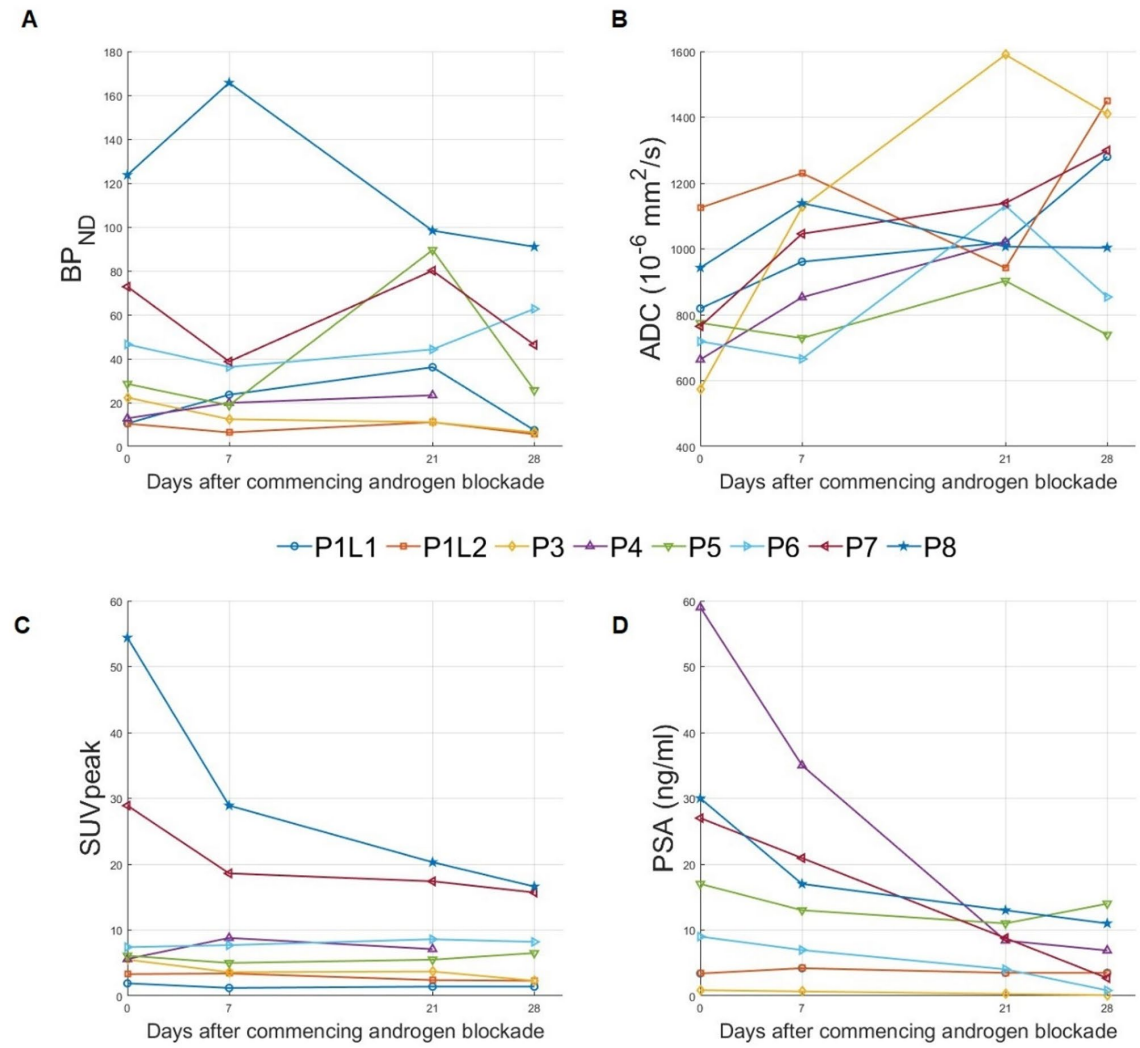
Longitudinal changes in [^68^Ga]Ga-PSMA-11PET/MR-derived imaging parameters in men with hormone-sensitive prostate cancer initiating bicalutamide treatment. (**A**) Non-displaceable PSMA binding potential (BP_ND_), (**B**) apparent diffusion coefficient (ADC), and (**C**) peak standardized uptake value (SUVpeak) for each assessed lesion. (**D**) Corresponding changes in plasma prostate-specific antigen (PSA) levels. Abbreviations: P = patient. Patient 1 had two lesions, labelled P1L1 and P1L2. Patient 2 remained PSMA PET-negative throughout the study and was excluded from imaging-based analyses

**Fig. 3:**
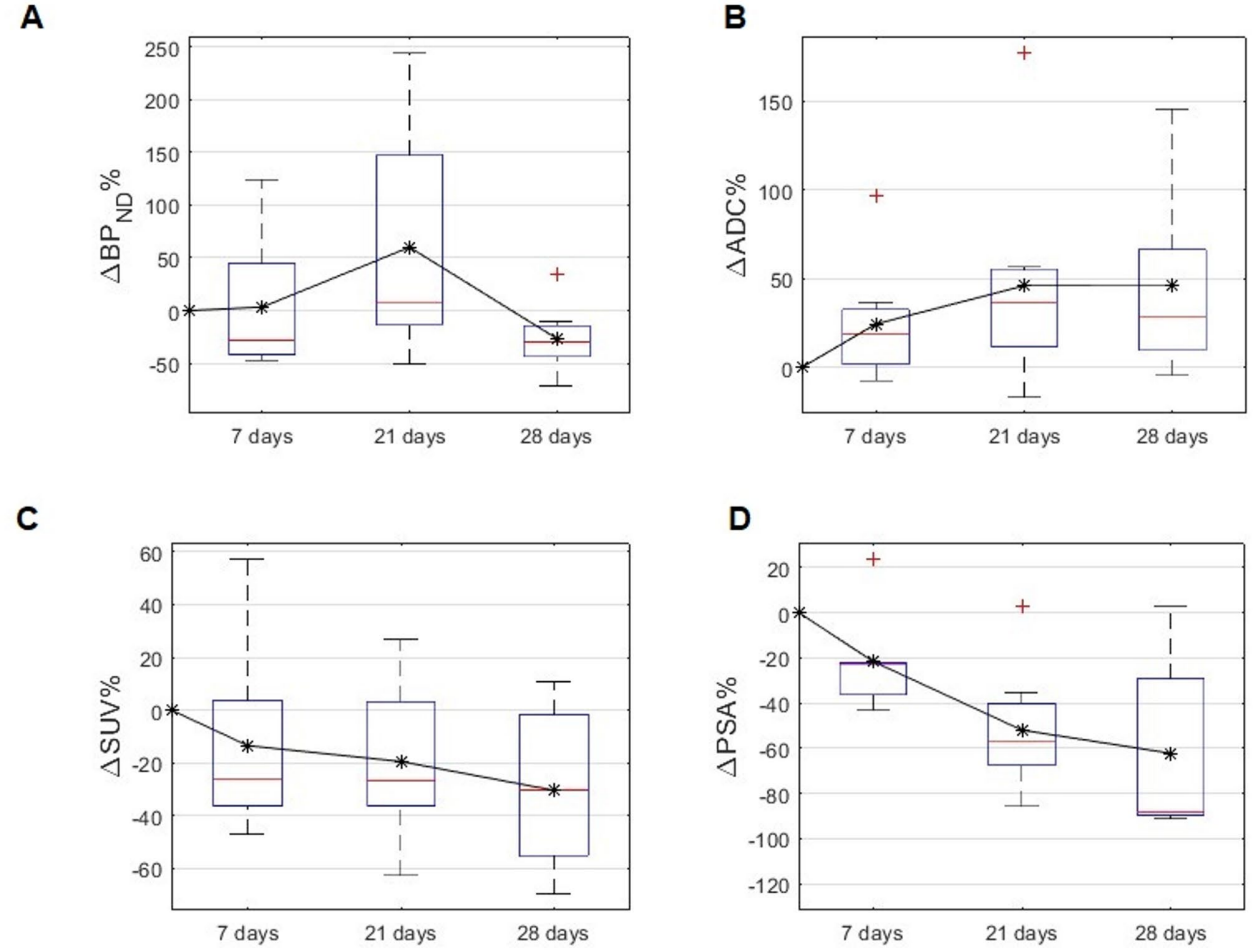
Boxplots depicting the group level median relative longitudinal fold change in (**A**) lesion non-displaceable PSMA binding potential (ΔBP_ND_%), (**B**) lesion apparent diffusion coefficient (ΔADC%), and (**C**) lesion peak standardized uptake value (ΔSUV%). **(D**) Boxplot of the longitudinal fold change in serum prostate specific antigen levels (ΔPSA%). Stars indicate the mean values at each time point

Table 1 presents the clinical characteristics and morphological disease descriptors of the study subjects at baseline, along with their corresponding changes throughout the study. A total of eight male patients were consecutively enrolled and completed the four serial [^68^Ga]Ga-PSMA-11PET/MR scans of the study, except for patient 4, who was unable to complete the final scan due to rescheduled surgery. All patients, except for patient 2, demonstrated [^68^Ga]Ga-PSMA-11lesion uptake. A total of 8 lesions were included in the data analysis. Figure 2 displays the individual longitudinal trajectories of ADC, BP_ND_ and SUVpeak for these lesions and the patient’s serum-PSA throughout the study duration. Figure 3 presents the corresponding group-level boxplots of their relative changes from baseline at each time point.

**Table 1:** Patient demographics and disease morphological and biochemical descriptors at baseline and after initiation of first-generation nonsteroidal antiandrogen treatment

Patient	Age at baseline (yr)	Disease presentation	Gleason score	Staging	Time from operation (months)	PSA baseline (ng/mL)	PSA w1 (ng/mL)	PSA w3 (ng/mL)	PSA w4 (ng/mL)	Change in PSMA-PET staging across study
1	59	Recurrence§	4 + 3	pT3b R0 N1 M0	43	3.4	4.2	3.5	3.5	No
2^¤^	48	Recurrence§	4 + 3	pT3a R1 N1 M0	4	5.2	4.4	2.5	2	No
3	76	Recurrence§	4 + 5	pT2 R0 N0 M0	20	0.9	-	-	0.1	No
4	55	Primary#	4 + 4	T2 N0 M0	-	59.0	35.0	8.4	6.9	No
5	68	Primary#	3 + 4	T3a N0 M0	-	17.0	13.0	11.0	14.0	No
6	72	Primary#	3 + 4	T2c N0 M0	-	9.0	7.0	4.0	0.8	No
7	84	Primary#	4 + 5	T4c N0 M0	-	27.0	-	-	2.7	No
8	57	Primary#	4 + 5	T3c N0 M0	-	30.0	17.0	13.0	11.0	No

§ = Recurrence post-prostatectomy, # = Primary diagnosis before any treatment, w = week. ¤ = remained PSMA PET-negative throughout the study

### PSA response to NSAA

Figure 2; Table 1 demonstrate a significant and rapid reduction in serum PSA levels in response to NSAA in all patients except patient 1, confirming a positive biochemical response. By the end of the study (day 28), the median ΔPSA% at the group level was − 88.3% (IQR: − 89.6% to − 29.1%), as shown in Table 2. Patient 1 exhibited stable PSA with temporal increase at week 1. Figure 2 also demonstrates that a positive biochemical response to NSAA was present even in patients exhibiting increases or stable values of lesion BP_ND_ throughout the study. As expected, the LME model shows that time on NSAA is a strong predictor for the decline in serum-PSA (estimate − 1.96, 95%CI: −2.58% to −1.34%, *p* = 0.0000004).

**Table 2:** Relative changes from baseline in the outcome variables at the group level following initiation of first-generation non-steroidal antiandrogen (NSAA) treatment

Days in NSAA	ΔBP_ND_%	ΔADC%	ΔSUV%	ΔPSA%
7	−28.1 (−41.4, 44.5)	19.1 (1.7, 32.6)	−26.3 (−36.2, 3.5)	−22.7 (−36.4, −22.2)
21	8.1 (−12.7, 148.0)	36.7 (11.7, 55.5)	−26.8 (−36.3, 3.2)	−56.7 (−67.2, −40.2)
28	−29.6 (−43.7, −14.2)	28.9 (9.5, 66.4)	−30.3 (−55.1, −1.7)	−88.3 (−89.6, −29.1)

Values are presented as median (interquartile range)

### Visual assessment of the serial PSMA-PET images and SUV dynamics during NSAA

**Fig. 4:**
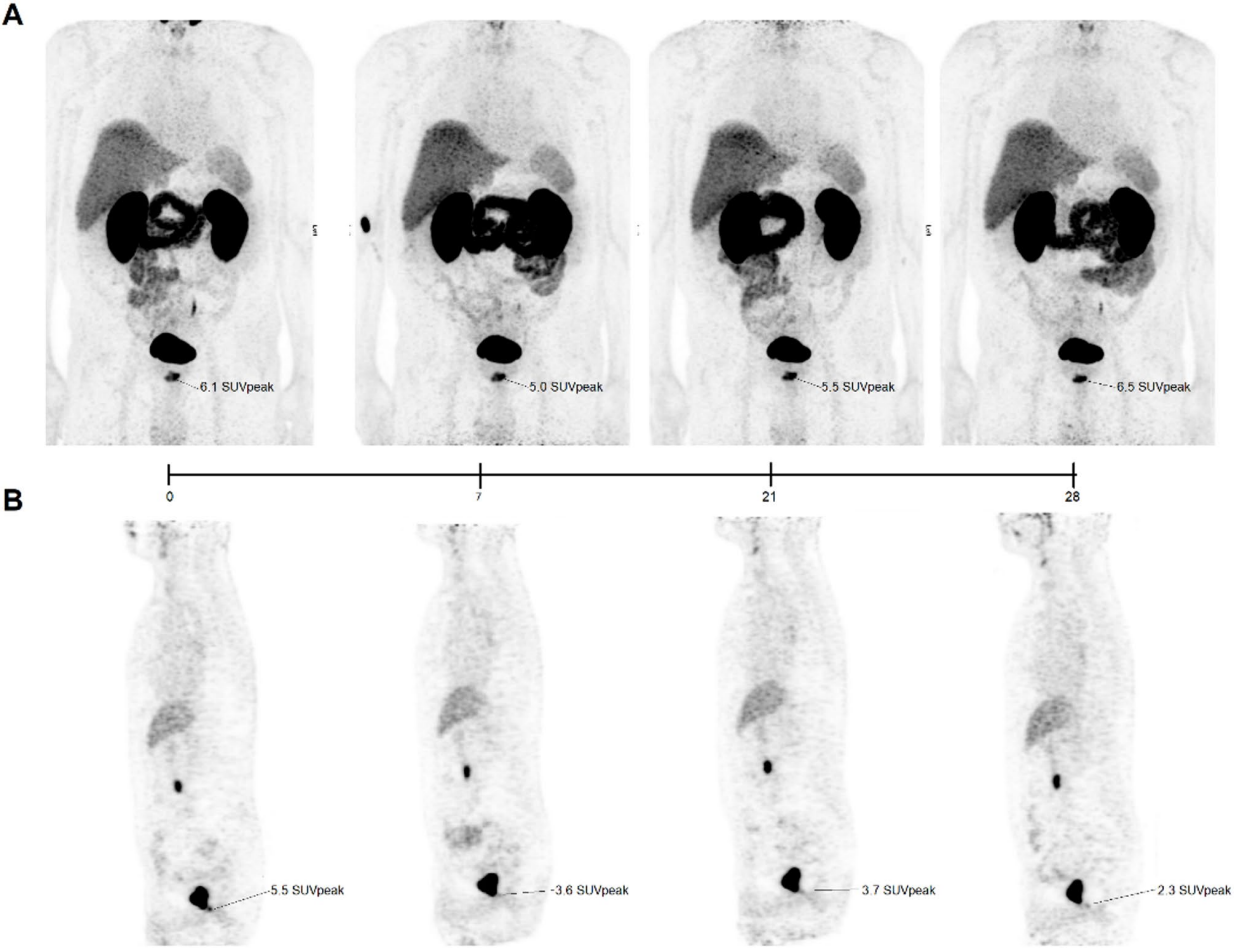
[^68^Ga]Ga-PSMA-11PET scans acquired 60 minutes after tracer administration over the study period (Day 0 to Day 28) in three representative individuals. (**A**) A 72-year-old man with newly diagnosed prostate cancer. (**B**) A 76-year-old man with local recurrence at the anastomosis site

Table 1 shows that no participants were upstaged from their baseline disease status during NSAA, with no new lesions appearing and no baseline lesions disappearing on [68Ga]Ga-PSMA PET/MR. Visual assessment of serial PET images revealed heterogeneous lesion-level responses. At the group level, semi-quantitative analysis of SUVpeak demonstrated a significant reduction over the study period, with a median SUVpeak fold change of − 30.3% (IQR: − 55.1% to − 1.7%) at day 28, and the LME model confirmed a significant linear decrease over time (estimate − 0.83%, 95% CI: − 1.44% to − 0.23%, *p* = 0.009). Despite this overall trend, several patients and lesions (patients 4–6 and lesion 2 in patient 1) showed increases in SUVpeak (Fig. 2), illustrating inter-patient and inter-lesion variability. Representative serial PET scans for patients 3 and 6 further highlight these heterogeneous responses based on SUV quantification (Fig. 4). Agreement analysis showed poor concordance between dynamic PET–derived ΔBP_ND_% and ΔSUV% based on static SUVpeak dynamics. ΔSUV% systematically underestimated changes in PSMA receptor density, as reflected by the positive bias (25.7%) and wide limits of agreement (−107.2% to + 158.6%). The concordance correlation coefficient was also low, 0.069 indicating that SUV metrics do not reliably capture nonlinear PSMA modulation. Detailed information of this agreement analysis is presented in the Supplementary Materials (Figure. S1).

### Temporal dynamics of lesion cellularity and PSMA expression under NSAA

Figures 2 and 3; Table 2 show heterogeneous and nonlinear temporal dynamics of lesion diffusivity (ΔADC%) and PSMA expression (ΔBP_ND_%) during NSAA treatment. At the group level, ΔBP_ND_% followed a biphasic course, with a reduction at day 7 of − 28.1% (IQR: − 41.4% to 44.5%), a transient increase at day 21 of + 8.1% (IQR: − 12.7% to 148.0%), and a subsequent decline at day 28 of − 29.6% (IQR: − 43.7% to − 14.2%). In contrast, ΔADC% increased progressively, consistent with reduced cellular density, with medians of + 19.1% (IQR: 1.7% to 32.6%), + 36.7% (IQR: 11.7% to 55.5%), and + 28.9% (IQR: 9.5% to 66.4%) at days 7, 21, and 28, respectively. This is consistent with a treatment-induced structural response characterised by a reduction in lesion-cellular density and improved diffusivity. Notably, the wide interquartile ranges, particularly for ΔBP_ND_% at day 21, reflect high inter-individual heterogeneity in PSMA modulation and structural response, which was also evident at individual-level (Fig. 2). While most lesions showed progressive increases in ADC, ΔBP_ND_% exhibited diverse individual patterns, including monotonic decline, biphasic, and S-shaped responses (Fig. 2), reflecting substantial inter-lesion variability. Individual LME time-modelling confirmed the significant linear increase in ΔADC% over time in NSAA (estimate 1.66, 95% CI: 0.73% to 2.59%, *p* = 0.001) and the nonlinear and non-monotonic trajectory for ΔBP_ND_%, with a significant cubic time effect, reflecting the presence of one inflection point (linear estimate − 5.63%, 95% CI: −21.08% to 9.81%, *p* = 0.46, quadratic estimate 1.11%, 95% CI: −0.24% to 2.46%, *p* = 0.10 and cubic estimate − 0.03%, 95% CI: −0.065% to −0.002%, *p* = 0.038). Extended LME-modelling of ΔBP_ND_%, including both time and ΔADC% as predictors (Table 3), supported this non-linear and non-monotonic trajectory and revealed a non-significant negative association between ΔBP_ND_% and ΔADC%. The agreement between the observed versus predicted ΔBP_ND_% using this extended LME model, is shown for each individual lesion trajectory in the Supplementary Materials (Figure S2).

**Table 3:** Main effects from the extended linear mixed-effects model examining the relationship between changes in lesion non-displaceable binding potential (ΔBP_ND_%), cellularity (ΔADC%), and time during first-generation non-steroidal antiandrogen treatment

Predictor	Estimate	SE	95% CI	*p*-value
Intercept	0.00	20.56	−42.27 to 42.27	1.0
Time	−3.29	7.36	−18.42 to 11.84	0.66
Time2	1.05	0.63	−0.26 to 2.35	0.11
Time3	−0.03	0.015	−0.06 to −0.002	0.033
ΔADC%	−0.55	0.28	−1.14 to 0.035	0.064

## Discussion

In this study, we investigated the short-term effects of first-generation NSAA treatment on PSMA expression in hormone-sensitive prostate cancer patients without bone metastases, using dynamic [^68^Ga]Ga-PSMA PET/MR imaging. Our results suggest that short-term NSAA induces a transient upregulation of PSMA expression, which subsides with continued androgen blockade.

Most of the lesions in this small cohort showed, compared to baseline, a decrease in PSMA PET tracer uptake and serum PSA concentrations and higher ADC values at end of ADT treatment (Table 2; Figs. 2 and 3), consistent with ADT treatment-induced reduction in tumour cellularity throughout the study period. This observation aligns with prior findings showing that prostate cancers with lower PSMA PET tracer uptake often exhibit higher ADC values, reflecting more infiltrative growth patterns or less densely cellular tumour regions [1]. Hence, under the assumption of constant PSMA PET tracer affinity, the temporary increase in ΔBP_ND_% observed at mid-treatment, despite declining cellularity, may indicate compensatory upregulation of PSMA in surviving tumour cells. This phenomenon is consistent with preclinical evidence suggesting that PSMA expression can transiently rebound in response to androgen suppression before ultimately decreasing with tumour regression [15, 16, 18, 20]. Although the negative association between ΔADC% and ΔBP_ND_% in the extended LME model did not reach conventional significance (*p* = 0.064), our results underscore the importance of accounting for tumour heterogeneity and adaptive mechanisms when interpreting imaging changes following short-term ADT, as PSMA-PET imaging and MR-diffusion metrics capture different underlying histopathological and cellular responses. Considering prior studies and our own findings, the temporal evolution of PSMA expression during ADT appears to follow a reproducible pattern with an early transient upregulation reaching a peak at around week 3, followed by a gradual decline and ultimately sustained downregulation beyond several months of treatment. This dynamic behaviour underscores the importance of considering the timing of PSMA PET imaging or radionuclide therapy in relation to ADT initiation. Further, we also observed notable inter-individual variability in the PSMA response dynamics (Tables 2 and 3; Figs. 2 and 3) underlying differences in tumour biology and pharmacodynamic response to NSAA. These patient-specific differences underscore the complexity of PSMA regulation under NSAA and suggest that group-level modelling may potentially miss heterogeneous temporal dynamics at the individual lesion or patient level. Such variability emphasizes the need for caution when interpreting the results of our study due to the limited number of patients included. However, the overall impact of short-term NSAA on the visual assessment of the [^68^Ga]Ga-PSMA uptake was minimal for all patients, suggesting that PSMA receptor upregulation may compensate cellular density reduction. This supports previous results that short term androgen blockade treatment does not impair PSMA-PET imaging in castration-sensitive PCa participants.

The poor agreement of SUVpeak with ΔBP_ND_ clearly highlights the limitations of static PET metrics in capturing complex, non-monotonic biological responses and supports the growing recognition that dynamic PET imaging provides a more sensitive assessment of treatment response in molecularly targeted therapies than static PET imaging endpoints.

### Study limitations

We recognize several limitations in this study. The primary limitation was the small sample size, which limits statistical power and possible model generalizability. While the mixed-effects modelling approach for ΔBP_ND_% could capture meaningful patterns for predicting PSMA upregulation, the large residual error of the model, 54.25%, and the wide 95%CI, 40.7% to 72.4%, reflect considerable uncertainty in the model estimates, likely due to the complexity of the model relative to the number of observations (Supplemental material Figure S2). Consequently, we were unable to stratify participants based on clinical subtypes (e.g., newly diagnosed vs. recurrent disease), age or Gleason -score, etc. which may impact the observed PSMA expression patterns. Ethical and resource constraints also limited the number of PET scans during the study, potentially missing more gradual temporal dynamics in PSMA receptor expression across the study duration. However, we believe the longitudinal within-patient design of the LME models in this study strengthens our conclusions and provides important preliminary data for future, larger confirmatory studies.

Although care was taken to ensure a consistent acquisition protocol, by allowing the patients to empty the bladder between scans, motion artifacts and image co-registration inaccuracies may have introduced variability in the quantitative measurements. In particular, internal pelvic organ motion—such as bladder filling or bowel peristalsis—during the 40 min long dynamic PET acquisition may have affected lesion localization in the dynamic scan. This is especially relevant for patients with biochemical relapse, all of whom presented with very small lesions in the pelvic area. Such motion can distort the time-activity curves used for BP_ND_ estimation and compromise ADC accuracy both through signal dropout and ghosting. Additionally, rigid co-registration to the 90-minute PET scan for each hospital visit may have caused minor spatial misalignments in the lesion VOI on the dynamic scan due also to internal motions. To minimize this effect, the VOI location in the dynamic PET scan was visually confirmed at each time point. Altogether, these technical issues should be carefully addressed in future studies using for instance advance machine learning strategies for motion correction and non-rigid image co-registration.

It should be emphasized that our findings relate specifically to short-term neoadjuvant ADT in hormone-sensitive prostate cancer. In contrast, in castration-resistant prostate cancer (CRPCa), the biological and imaging effects of ADT may differ substantially due to tumour evolution, androgen receptor pathway alterations, and increased heterogeneity. Recent studies in the advanced CRPCa setting, including those investigating PSMA-targeted radioligand therapy such as [177Lu]Lu-PSMA-617, further illustrate how tumour genomic instability and variable PSMA expression impact radionuclide treatment response and PET imaging readouts [25–27]. Hence, the ADT modulating effects on PSMA expression described in this work cannot be directly extrapolated to the CRPCa setting, and further studies are required.

## Conclusions

To our knowledge, this is the first prospective longitudinal study using dynamic [^68^Ga]Ga-PSMA-11 PET/MR to explore short-term changes in PSMA expression under NSAA in hormone-sensitive prostate cancer patients. Our results suggest that early PSMA expression may follow a biphasic trajectory, with transient upregulation potentially balancing out concurrent reductions in tumour cellularity. These dynamics, in conjunction with the use of static SUV metrics alone, may explain previous discrepancies in earlier clinical studies. Furthermore, our data indicate that staging with PSMA PET tracers is not compromised by early NSAA and that short-term PSMA upregulation could be predicted with quantitative PET imaging. This highlights the potential use of dynamic PET imaging to monitor treatment response and optimize personalized therapeutic strategies in this patient group. Altogether, these insights may have clinical implications for possible therapeutic interventions using PSMA-targeted radioligands in combination with androgen blocking agents in hormone-sensitive PC patients.

## Supplementary Information

Supplementary Material 1.

## Data Availability

Data are available on reasonable request.
