# Supplementary figures and images for: The impact of short-term non-steroidal androgen antagonist therapy on PSMA expression and tumor cellularity studied with dynamic [68Ga]Ga-PSMA-11 PET/MR in hormone-sensitive prostate cancer patients, a preliminary longitudinal prospective study
Source: EJNMMI Res. 2025 Oct 2;15:127. doi: 10.1186/s13550-025-01328-1 (PMC12491120; doi:10.1186/s13550-025-01328-1)

**Supplementary Materials**

**Figure S1**

***
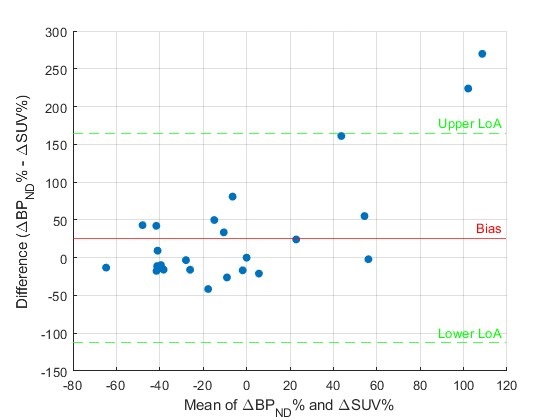
***

**Figure S2** ***
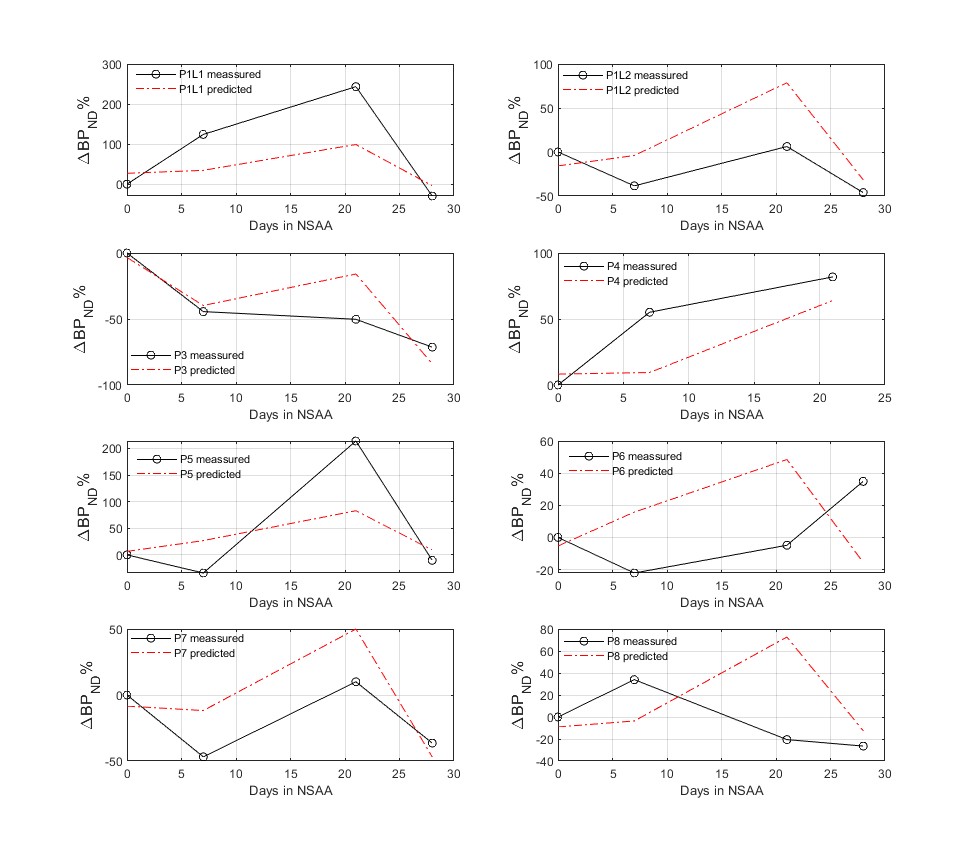
***

Supplement: Supplementary file 1 — Supplementary Material 1. [file 13550_2025_1328_MOESM1_ESM.docx]
